# Laboratory data on long-term sealing behaviors of two water-swelling materials for shield tunnel gasket

**DOI:** 10.1016/j.dib.2020.105609

**Published:** 2020-04-22

**Authors:** J.S. Tan, S.L. Shen, A. Zhou, Z.N. Wang, H.M. Lyu

**Affiliations:** a Department of Civil Engineering, School of Naval Architecture, Ocean, and Civil Engineering, Shanghai Jiao Tong University, 800 Dong Chuan Road, Minhang District, Shanghai 200240, China; b Key Laboratory of Intelligent Manufacturing Technology (Shantou University), Ministry of Education, and Department of Civil and Environmental Engineering, College of Engineering, Shantou University, Shantou, Guangdong 515063, China.; c Discipline of Civil and Infrastructure, School of Engineering, Royal Melbourne Institute of Technology (RMIT), Victoria 3001, Australia; d State Key Laboratory of Internet of Things for Smart City, University of Macau, Macau S.A.R., China

**Keywords:** Swelling behavior, Storage modulus, Micro-damage morphology

## Abstract

This article provides comprehensive experimental data of two water-swelling materials, water swelling rubber (WSR) and water-swelling polyurethane (WSP). Swelling tests, Dynamic Mechanical Analyzer (DMA) and Scanning Electron Microscope (SEM) were performed. Sealing properties of WSR and WSP were characterized by the data of swelling ratios (S_w_ and S_a_), storage moduli (E') and images of micro-damage morphologies. These data can be useful for the prediction of the long-term waterproof performance of water-swelling materials and provide reference for material selection. The data presented herein was used for the article, titled “Laboratory evaluation of long-term sealing behaviors of two water-swelling materials for shield tunnel gasket” [1].

Specifications Table

**Table:** 

Subject	Material and Engineering
Specific subject area	Waterproof materials, mechanical property
Type of data	Table, Figure
How data were acquired	Swelling test: Self designed indoor experiment DMA: Q850 SEM: Sirion 200 microscope
Data format	Raw and analyzed
Parameters for data collection	The working conditions of compression loads and saline environments are set in all experiments. Four compression loads of 1, 2, 3 and 4 MPa are set for WSR samples. Three compression loads of 1, 1.5 and 2 MPa are set for WSP samples. Distilled water, artificial seawater, and concentrated artificial seawater are set as experimental immersion solutions both for WSR and WSP.
Description of data collection	The data of swelling behaviors, storage moduli, and micro-crack morphology of WSR and WSP were harvested from swelling experiments, Dynamic Mechanical Analysis (DMA) and SEM, respectively. The raw data can be read directly by dial gauge or data taker of experimental instrument.
Data source location	Civil Engineering Laboratory, Shanghai Jiaotong University, Shanghai, China
Data accessibility	Data provided in the article are accessible to the public
Related research article	Ahthors's name: Jishuang Tan; Shui-Long Shen; Annan Zhou; Zenian Wang; Haimin Lyu; Title: Laboratory evaluation of long-term sealing behaviors of two water-swelling materials for shield tunnel gasket; Journal: Construction and building materials DOI: https://doi.org/10.1016/j.conbuildmat.2020.118711

## Value of the data

• The comprehensive data can be used for prediction of the long-term waterproof performance of WSR and WSP
• Waterproof materials, mechanical property
• The data of swelling test are valuable for understanding the long-term swelling behaviors of WSR and WSP
• The data about storage moduli provides accurate comparison of WSR and WSP, demonstrating the potential application of DMA to measure the dynamic mechanical properties for low strength material
• The data provides reference for material selection of WSR and WSP

## 1 Data description

**Fig. 1:**
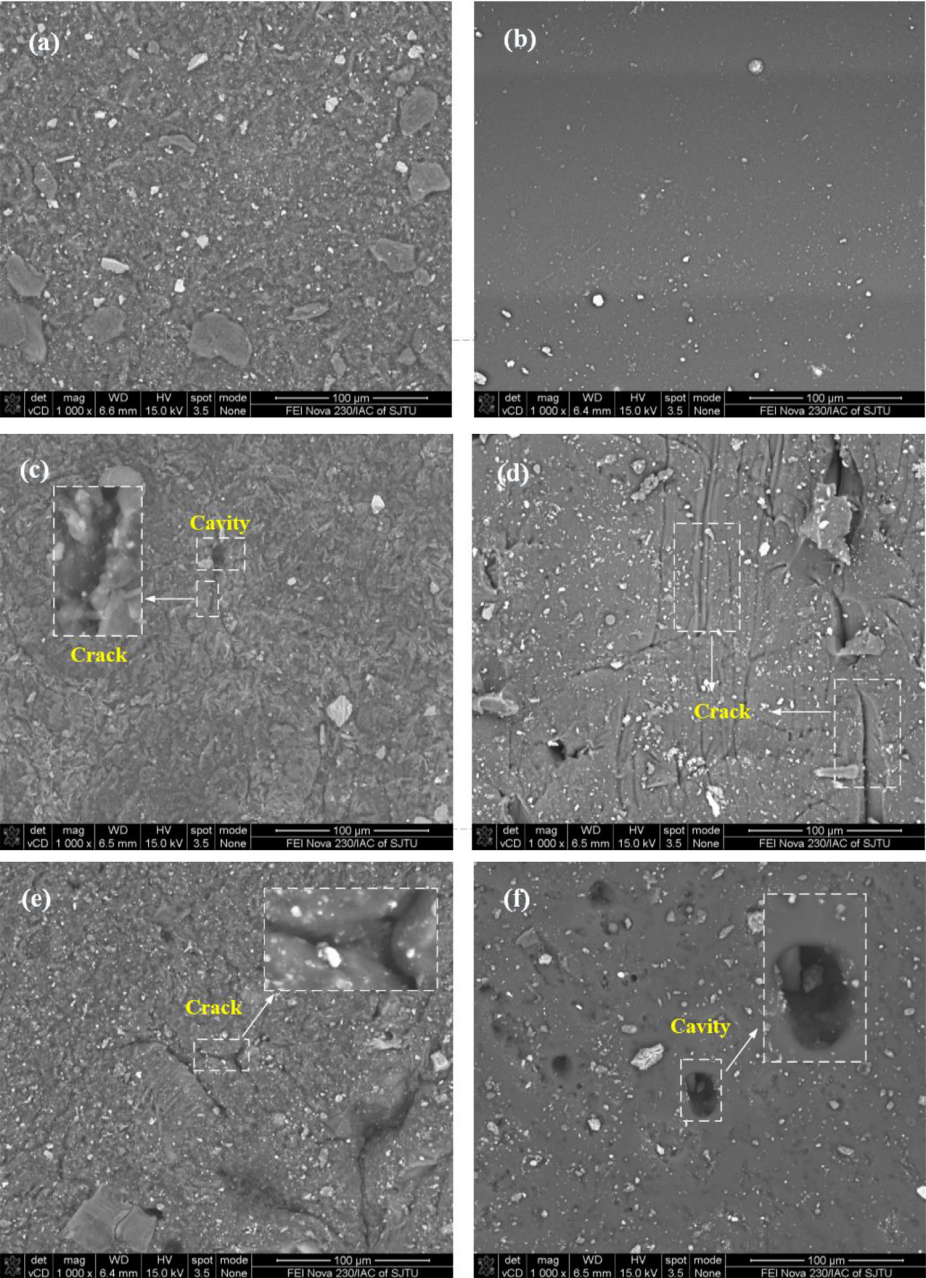
SEM images of (a) original WSR specimen; (b) original WSP specimen; (c) WSR under compression load of 1MPa; (d) WSP under compression load of 1MPa; (e) WSR immersed in concentrated artificial seawater; (f) WSP immersed in concentrated artificial seawater;

The data consists of swelling ratio (S_a_ and S_w_), storage moduli (E’) and SEM images, which is used to access the sealing capacity of water swelling rubber (WSR) and water-swelling polyurethane (WSP). Table 1 and Table 2 present the swelling ratio (S_a_ and S_w_) of specimens, respectively. Table 3 and Table 4 present the storage moduli of specimens under compression and in saline environments, respectively. Fig. 1 shows the morphology of original specimens and the micro-crack morphology of specimens under compression and in saline environments.

**Table 1:** The axial swelling ratio S_a_ (%) of the WSR and WSP specimen under different compression loads.

	WSR				WSP		
Time(h)	Compression load (MPa)						
	1	2	3	4	1	1.5	2
0.25	0.87						
0.3		0.17	0.16	0.17			
0.5					3	3.5	5
0.75	1.53						
1.3833		0.83	0.33	0.33	8.5		
1.75	2.37					5	
2.8		1.17	0.83	0.5	17		
4.5					21.5		11
6.25	6.2				29	23	
8.9667		4.5	2.16	1.83			
9.75	7.37						
10.9667		5	3.16	2.17		39	
11.75	7.7				46		39.5
23.25	9.2				69.5	75	62.5
29.29		7.5	6	5			
35					80		70
47.25	11.37				87		73
50.13		9.83	8.33	7.5		80.5	
57.25	12.03						
60.75	12.7				90		73
61.25	13.87						
73.63		11.83	10	8.67	92	84	73.5
85.25	15.37					86.5	
97.63		14.67	11.5	10.17	91.5		73.5
109.63		15.83	12.5	11.5		90	
118.25	16.53				93		68.5
121.63		16.83	13.33	11.83			
137.75	17.87	17.5	13.5	13.17		92	
146.5					93.5		69
161.75	19.03					90	
177.63		20.33	16.33	14.33			
182.75	20.37					88.5	
193.63		20.83	16.83	15.17	94		68.5
206.75	21.53					89.5	
215.63		22	17.5	15.67	91		65
230.25	22.87						
263.32		24	19.16	16.5	87.5		61
278.25	23.87					86.5	
287.63		24.17	19.66	16.83	85		59
329.25	24.53					83.5	
377.25	25.03						
388.13		25.5	20.66	16.67			
395					84	84	58.5
425.25	25.37						
447.5		25.4	21.21	15.98	81		58
490					78.5		53.5
521.25	25.87						
555.5	25.7					77.5	
585.5		25.2	21.55	16.28			
617.25	25.53	25.1	21.82	16.33	73.5	73	49
652.13		25	22	15.5	75		50.5
689.25	25.37					72	
700.13		24.83	21.83	15	75.5		50.5
761.25	25.2				76	74.5	51
796.13		24.33	21.16	14.17			
809.25	24.87				73		48
844.13		24	20.83	13.67			
857.25	24.53				70.5		45.5
875					68	72	43
892.63	24.37	23.67	20.66	13.56

**Table 2:** The free swelling ratio S_w_ (%) of the WSR and WSP specimen in different saline environments

	WSR			WSP		
Time(h)	Saline environment					
	Distilled water	Artificial seawater	Condensed artificial seawater	Distilled water	Artificial seawater	Condensed artificial seawater
12	61.04	32	40.54	51.77	61.55	45.623
36	126.81	43.29	43.75	88.85	86.92	65.313
60	162.04	45.79387	53.25448	123.30552	95.6434	85.40078
108	197.75429	46.70615	53.25448	126.75379	115.09923	89.10448
156	215.61143	47.70615	49.80621	126.75379	118.94538	89.10448
204	219.18286	50.55231	46.35793	128.9071	118.94538	92.80819
252	226.32571	54.39846	46.35793	130.20207	118.94538	103.9193
300	217.61143	50.55231	42.90966	119.85724	103.56077	92.80819
348	210.46857	50.55231	36.0131	119.85724	99.71462	92.80819
420	203.89714	46.70615	36.0131	116.40897	95.86846	89.10448
468	199.32571	46.70615	32.56483	116.40897	95.86846	85.40078
516	197.18286	43.64	32.56483	116.40897	92.02231	81.69707
564	195.46857	42.86	32.56483	112.96069	88.17615	81.69707
636	192.61143	42.86	32.56483	112.96069	88.17615	81.69707
708	189.61143	42.86	29.11655	109.51241	76.63769	74.28967
804	184.75429	39.01385	29.11655	109.51241	76.63769	70.58596
888	182.61143	35.16769	25.66828	109.51241	72.79154	63.54893

**Table 3:** The storage moduli E’ (kPa) of WSR and WSP specimen under different compression loads

	WSR				WSP		
Time (h)	Compression load (MPa)						
	1	2	3	4	1	1.5	2
200	34	133	240	273	126	149	245
400	47.1297	210.149	323.731	385.458	217.931	281.667	357.092
600	51.7	296	470	559	171.4	267.8	327
900	52.4628	357.888	482.468	571.6759	170.221	238.321	322.228

**Table 4:** The storage moduli E’ (kPa) of WSR and WSP specimen under different saline environments

	WSR			WSP		
Time (h)	Saline environment					
	Distilled water	Artificial seawater	Condensed artificial seawater	Distilled water	Artificial seawater	Condensed artificial seawater
12	208.291	834.698	1050	666.812	923.082	1108.96
81	55.6014	724.589	780.787	581.333	854.465	965.465
264	49.3977	619.246	688.424	519.535	709.145	955.899
552	25.7865	160.148	412.136	431.081	620.948	806.607
912	16.29114	74.5364	325.564	207.599	447.54	491.48

## 2 Experimental design, materials and methods

### 2.1 Design, materials and methods

WSR and WSP are the main waterproof materials for the hydrophilic gasket of shield tunnel [1], [2], [3]. There are two main methods for the preparation of water swelling materials: rubber blending method and chemical polymerization method. Water swelling rubber (WSR) belongs to the former, which is prepared by blending and vulcanizing water absorbent resin with carrier rubber; water swelling polyurethane (WSP) belongs to the latter, which is prepared by chemical polymerization of low molecular organic body [4].

To investigate the sealing capacities, the macroscopic dynamic properties and micro-damage morphologies of the WSR and WSP, laboratory tests-including the swelling test, dynamic mechanical analyzer (DMA) and SEM were performed with different compression loads and saline environments. The saline environments in this experiment consists of distilled water, artificial seawater and condensed artificial seawater with double ion concentration. The configuration of artificial seawater can be found in Garcia et al. [5].

### 2.2 Measurement data

In this data article, the axial swelling ratio (S_a_) and free swelling ratio (S_w_) [1] are used to characterize the swelling capacity of WSR and WSP.

Table 1 shows the variation of S_a_ with time of the WSR and WSP specimen under different compression load.

Table 2 shows the free swelling ratio (S_w_) with time of the WSR and WSP specimen in different saline environments.

The storage moduli E’ (kPa), which is typically related to Young's modulus [6], are used to characterize the dynamic mechanical properties of WSR and WSP. Table 3 and Table 4 lists the E’ value under different compression loads and saline environments.

SEM images present micro-crack morphology of WSR and WSP, which is helpful for the assessment of damage characteristics under long-term compression or immersion in different solutions [7]. Fig. 1 shows the SEM images of WSR and WSP specimen under compression loads and immersed in different saline environments after 900 hours.

## Appendix Supplementary materials

Image, application 1

Image, application 2
